# Predicting tropospheric nitrogen dioxide column density in South African municipalities using socio-environmental variables and Multiscale Geographically Weighted Regression

**DOI:** 10.1371/journal.pone.0308484

**Published:** 2024-08-08

**Authors:** Sphamandla N. Hlatshwayo, Solomon G. Tesfamichael, Mahlatse Kganyago

**Affiliations:** Department of Geography, Environmental Management and Energy Studies, University of Johannesburg, Johannesburg, South Africa; Federal University of ABC, BRAZIL

## Abstract

Atmospheric nitrogen dioxide (NO_2_) pollution is a major health and social challenge in South African induced mainly by fossil fuel combustions for power generation, transportation and domestic biomass burning for indoor activities. The pollution level is moderated by various environmental and social factors, yet previous studies made use of limited factors or focussed on only industrialised regions ignoring the contributions in large parts of the country. There is a need to assess how socio-environmenral factors, which inherently exhibit variations across space, influence the pollution levels in South Africa. This study therefore aimed to predict annual tropospheric NO_2_ column density using socio-environmental variables that are widely proven in the literature as sources and sinks of pollution. The environmental variables used to predict NO_2_ included remotely sensed Enhanced Vegetation Index (EVI), Land Surface Temperature and Aerosol Optical Depth (AOD) while the social data, which were obtained from national household surveys, included energy sources data, settlement patterns, gender and age statistics aggregated at municipality scale. The prediction was accomplished by applying the Multiscale Geographically Weighted Regression that fine-tunes the spatial scale of each variable when building geographically localised relationships. The model returned an overall R^2^ of 0.92, indicating good predicting performance and the significance of the socio-environmental variables in estimating NO_2_ in South Africa. From the environmental variables, AOD had the most influence in increasing NO_2_ pollution while vegetation represented by EVI had the opposite effect of reducing the pollution level. Among the social variables, household electricity and wood usage had the most significant contributions to pollution. Communal residential arrangements significantly reduced NO_2_, while informal settlements showed the opposite effect. The female proportion was the most important demographic variable in reducing NO_2_. Age groups had mixed effects on NO_2_ pollution, with the mid-age group (20–29) being the most important contributor to NO_2_ emission. The findings of the current study provide evidence that NO_2_ pollution is explained by socio-economic variables that vary widely across space. This can be achieved reliably using the MGWR approach that produces strong models suited to each locality.

## 1. Introduction

Air pollution is a significant challenge affecting populations globally with more impact observed in developing countries [1]. Due to emissions from electricity generation, transportation, and residential fossil fuel burning, most of the world’s population lives in areas where air pollution levels exceed the World Health Organization’s health-based air quality limits [1]. Exposure to air pollution poses a major threat to human health and is associated with illnesses such as cancer, stroke, asthma and heart attacks [2]. In addition, air pollution is known to contribute to environmental problems, including climate change and acidification of soil and water bodies [3, 4]. Nitrogen dioxide (NO_2_) is one of the main pollutants distributed across the atmosphere [5–7]. Research on the global trends of NO_2_ distribution suggests that tropospheric NO_2_ column density has been slightly increasing over time [8–10], although the opposite trend was observed during the lockdown forced by the coronavirus (COVID-19) pandemic that broke out in 2019 [11, 12].

The column density and distribution of NO_2_ can be mediated by environmental variables that may serve as sinks or sources of atmospheric pollutants. Vegetation, for example, uses NO_2_ to create amino acids, which stimulate plant growth, resulting in the removal of the compound from the atmosphere [13, 14]. A similar action by indoor plants decreases the indoor levels of NO_2_ that may arise from the burning of biomass, paraffin, coal and wood [15]. Another environmental factor that affects atmospheric NO_2_ level is land surface temperature (LST), which refers to the radiative temperature of the earth’s surface as a result of solar radiation [16]. The theoretical influence of LST on NO_2_ is acknowledged as high NO_2_ levels are observed under low-temperature conditions due to increased anthropogenic activities [17, 18]. The formation of NO_2_ depends more on direct source emission compared to photochemical processes, hence NO_2_ being high in winter compared to summer [18, 19]. High levels of NO_2_ during winter indicate energy demand observed by the increased pollution from household combustion and other anthropogenic activities [20]. Aerosol Optical Depth (AOD), which refers to a measurement of particles suspended in the atmosphere, also plays an important role in the levels of NO_2_ [21]. Aerosols are mostly produced at the surface of the earth because of various natural occurrences such as wind-borne dust, sea spray, volcanic debris and biological aerosols, as well as anthropogenic activities, industrial, agricultural-related dust, fossil fuel combustion and biomass burning [22].

Social factors such as population density can also affect atmospheric NO_2_ column density [23, 24]. For example, research has shown that most air pollution is concentrated in metropolitan areas with high population density [23–25]. It is also important to acknowledge that high air pollution can occur in rural and peri-urban areas due to domestic biomass burning and cross-boundary transportation of atmospheric particles [26]. The impact of population density on air pollution is mainly driven by increased energy consumption for households, industrial activities and transportation to meet population demand [23, 27]. Gender may also play a significant role in the emission of atmospheric pollutants, with women responsible for most household activities, likely to produce higher NO_2_ emissions than men. This also suggests that women and children have a higher exposure to the effects of NO_2_ emission than men [28]. Literature on the influence of gender on NO_2_ using remote sensing has mostly focused on the health effects of exposure to air pollution amongst men and women and the direct polluters are still unclear [29, 30]. Although the influence of age on atmospheric pollution is uncertain, epidemiological research shows that children and the elderly are highly vulnerable to the effects of air pollution and cardiorespiratory disorders because of their weakened immune systems [31]. Moreover, air pollution is associated with acute lower respiratory diseases in children less than the age of five years [32].

Developing countries tend to be the most affected by air pollution leading to their poor health and environmental conditions [28, 33–35]. Specifically, limited focus has been placed on the plight of air pollution in Africa leading to rampant socio-economic and environmental problems affecting the health of the population [34]. A recent statistic from Africa shows that, in 2019, indoor and ambient air pollution accounted for 697,000 and 394,000 deaths, respectively [35]. The recorded deaths induced by ambient air pollution were linked specifically to non-communicable diseases such as heart disease and chronic respiratory disease [35]. Industrialization, specifically in metropolitan areas of developing countries, is also contributing to air pollution and, thus health and environmental hazards [33, 36].

The correlation of NO_2_ levels with socio-environmental variables is crucial to inform the required levels that benefit the environment and human health. This can be achieved by integrating spatial analysis and remote sensing that provides a quick and unbiased synoptic view of spatial variations of atmospheric pollution [8, 20, 37–40]. Zhu [37] predicted NO_2_ in Chengdu, China, using Ozone Monitoring Instrument (OMI) meteorological and land use types and a Random Forest regression. The study found that NO_2_ column density was higher in areas where anthropogenic activities were high. Similarly, in the Jiangsu province of China, [41] found similar results on the influence of temperature in increasing NO_2_ levels in urban regions indicating the impact of factors such as industrial activities and population density on the pollution levels. Swartz [20] modelled long-term trends of atmospheric gases, including NO_2_, in Mpumalanga and Limpopo, South Africa, using meteorological variables, population growth and Multiple Linear regression. The findings showed increased NO_2_ column density with population growth, suggesting human-induced pollution activities in the regions. Moreover, a positive correlation between relative humidity and NO_2_ was observed by the research, explaining the seasonal variation in NO_2_ column density [20]. Based on a systematic review of remote sensing-based works focusing on the World Health Organization European Region, [42] found that social variables, including economic status and ethnicity, were linked to NO_2_ levels. Specifically, the exposure to NO_2_ was less in high-income neighbourhoods compared to lower-income neighbourhoods. Moreover, [42] found that men had less exposure to NO_2_ than women.

South Africa is renowned for its coal deposits and heavy reliance on it for power generation; as a result, NO_2_ represents one of the major pollutants in the country [43]. Besides electricity generation, other anthropogenically induced sources of NO_2_ pollution in South Africa include transportation, fuel combustion, biomass burning and indoor air pollution [44–46]. Due to rising urbanization, waste burning has become a source of environmental and air pollution in South Africa contributing to atmospheric pollutants like NO_2_ [45]. A continuous increase in population density, economic growth and urbanization necessitates a greater number of vehicles, leading further to NO_2_ emissions from fuel combustion [44].

The spatial distribution of NO_2_ across South Africa exhibits a high NO_2_ pollution in municipalities located in the northeastern region compared to the rest of the country due to industrial activities and electricity generation in that region [47]. Variation in household energy consumption across South Africa is attributed to income inequality, geographical and social diversity and economic volatility [48, 49]. Income is a major determinant of access to electricity, with poor households having no access to electricity [49, 50]. There is a need to assess if a comprehensive list of socio-environmental variables can be used to predict atmospheric NO_2_. A recent research by [47] investigated the distribution of NO_2_, SO_2_ and Sulphates (SO_4_) concentrations across South Africa using AOD (elevated smoke and polluted dust) and wind data. The study focused on trend analysis of the pollutant observations without incorporating explanatory variables. In addition, [47] used only environmental data (wind) to qualitatively explain pollution distribution. Therefore, using various socio-environmental variables as predictors within a statistical model allows for a more comprehensive analysis and understanding of the health implications of pollutants [2, 28]. Considering that a majority of South African households across the country live below the poverty level and rely on alternative energy sources that contribute to NO_2_ pollution necessitates the inclusion of social factors to predict the pollution [51]. The objective of this study was to predict annually derived tropospheric nitrogen dioxide (NO_2_) column density using socio-environmental variables and Multiscale Geographically Weighted Regression at municipal scale in South Africa. The study is significant as, firstly, it represents the first national-scale assessment in South Africa and secondly, such a study provides valuable information for a rapid municipality-level expectation of atmospheric NO_2_ pollution that can be exploited for decision-making at both the local and national levels.

## 2. Methods

### 2.1 Study area

**Fig 1:**
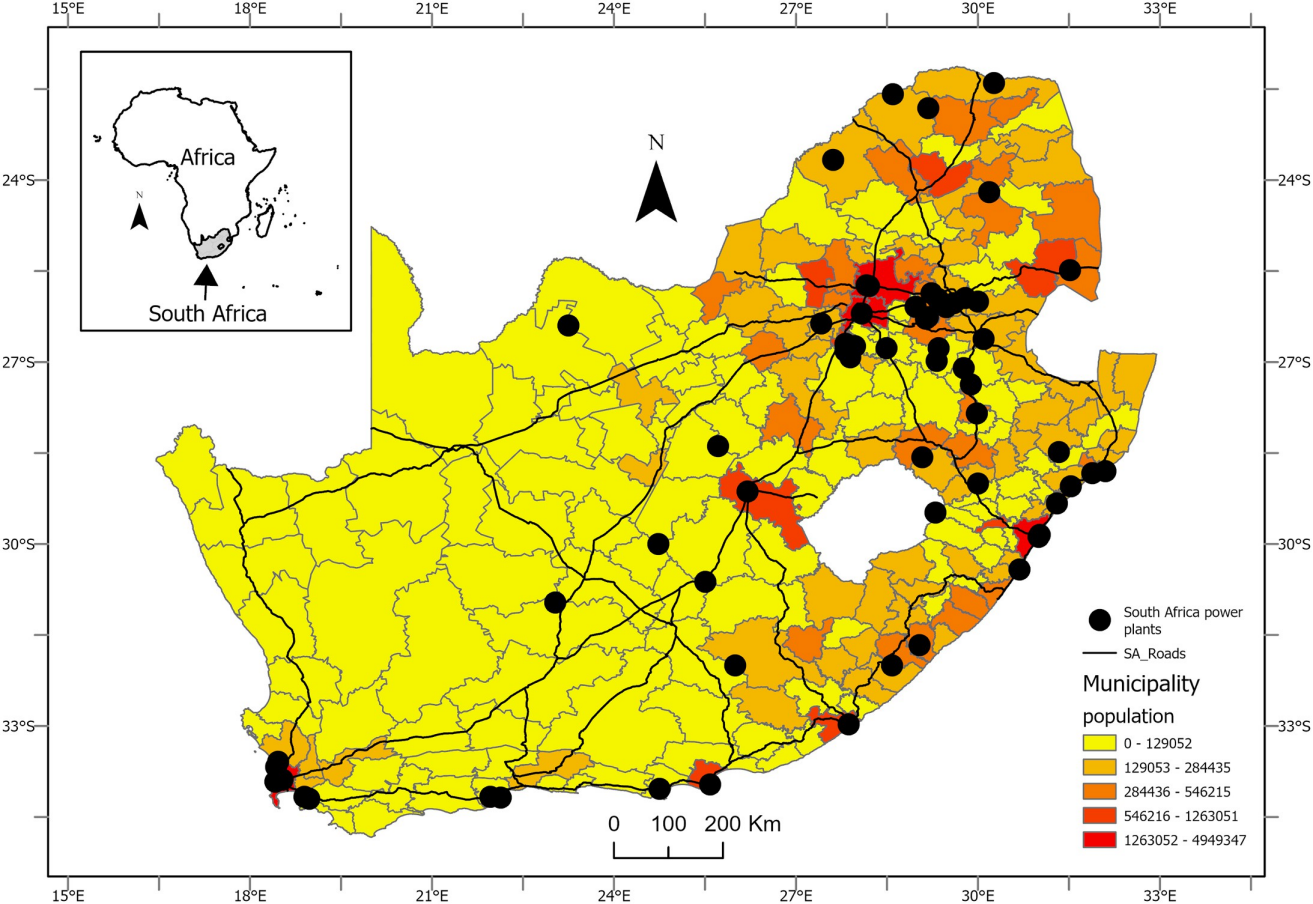
Study area showing power stations, major roads and municipality-level population distribution of South Africa.

The present study covers the entire South Africa (Fig 1), which has an area of 1 219 090 km^2^ and has a coastline extending roughly 3200 km [52]. The country is divided into nine provinces subdivided into 213 local municipalities, including eight metropolitan district-level municipalities [53]. According to mid-year estimates for 2022, the country had a population of 60 604 992 with a life expectancy of 59 and 65 years for men and women, respectively [54]. Most of the local municipalities in South Africa are poverty-stricken, with rural areas being poorer than urban areas [51, 55]. It is believed that three provinces found in the north and north-central parts of the country (i.e., Mpumalanga, Limpopo and Gauteng) are the leading sources of anthropogenic NO_2_ pollution in South Africa due to the power plants found in these provinces [47].

### 2.2 Description of data

#### 2.2.1 Environmental data

**Fig 2:**
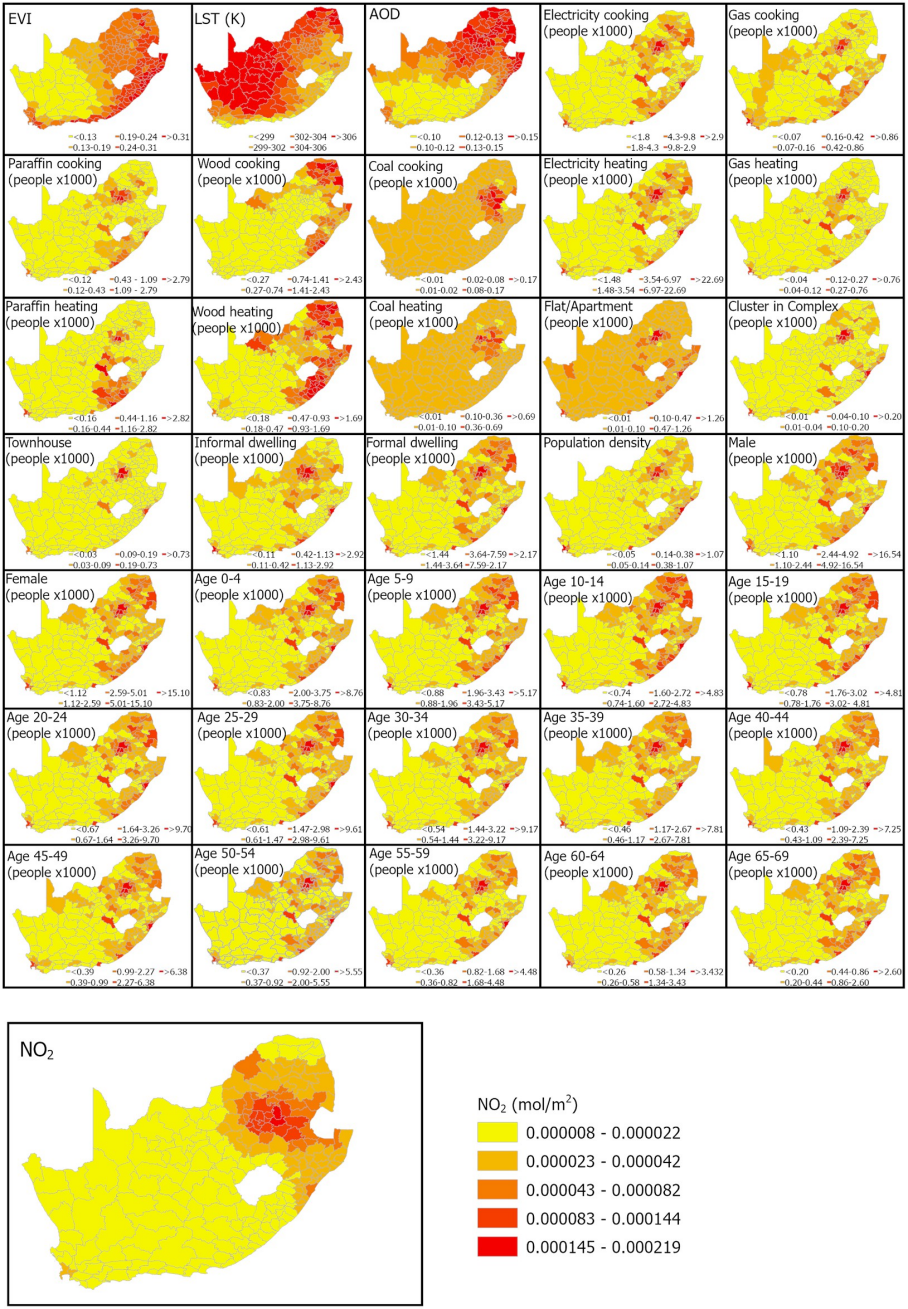
The spatial distributions of socio-environmental predictors and NO_2_ (variable to predict) by municipalities. The environmental variables (EVI, LST, AOD) and NO_2_ represent average values from December 2018 to November 2019. The legends of all social variables show the count of people divided by 1000.

NO_2_ data acquired by the Sentinel-5P satellite sensor was downloaded from the Copernicus Open Data Hub platform (https://scihub.copernicus.eu/, (Accessed 06 February 2023). Eight spectral bands, including the ultraviolet and visible light (270–495 nm), near-infrared (675–775 nm), and shortwave IR (SWIR) (2305–2385 nm) spectrum, are acquired by Sentinel-5P equipped with TROPOMI sensor [56]. The satellite was launched on October 13, 2017, to monitor and forecast the global climate and measure atmospheric air quality factors with high spatial and temporal resolutions [56]. Moreover, data from Sentinel-5P provide continuous spatial coverage with a 3.5 x 5.5 km spatial resolution. For the present study, the daily-averaged NO_2_ column density dataset for December 2018 to November 2019 was computed using the Google Earth Engine (GEE) platform and downloaded for further processing. Then, an average value was calculated for each municipality from the resultant annual NO_2_ data. The mean municipality-level NO_2_ distribution shows higher column density in the eastern and northeastern parts of South Africa (Fig 2).

The environmental parameters, i.e., EVI, AOD and LST, were retrieved from Moderate Resolution Imaging Spectroradiometer (MODIS) satellite data for the period between December 2018 to November of 2019. The GEE was used to download this dataset as image collections, which are ingested from the National Aeronautics and Space Administrations (NASA) Land Processes Distributed Active Archive Center (LP DAAC) ((https://lpdaac.usgs.gov/products/mod13a2v006/, (Accessed 06 February 2023)). The MODIS EVI (MOD13A2), LST (MOD11A1) and AOD products had a spatial resolution of 1 km. MOD13A2 is a 16-day composite based on the best pixel value characterized by low cloud cover and view angle as well as the highest EVI value within the 16-day period [57]. On the other hand, the MOD11A1 product is available daily and built using the daily LST pixel values acquired using the generalized split-window technique under clear-sky conditions [58]. Finally, the AOD product (MCD19A2) was acquired from the MODIS Multi-Angle Implementation of Atmospheric Correction (MAIAC), which is generated by integrating time series analysis and a combination of pixel- and image-based processing; MAIAC provides accurate spectral reflectance that is used for cloud identification, aerosol retrievals, and Earth feature extraction [59]. Accuracy in retrieving AOD data is achieved by the post-processing stage, where several filters are employed to detect residual clouds and smooth the noise introduced from the grid from uncertainties [59]. The MODIS MAIAC AOD products are derived using the blue (0.47 μm) and green (0.55 μm) spectral bands; in this study, the blue spectral band was used as this is more sensitive to aerosol variations in the atmosphere [59].

Each of the products described above was averaged per annum using the GEE platform. Subsequently, the annually-averaged environmental parameters were spatially averaged by municipal boundaries, and the period was consistent with NO_2_ column density data. A summary of statistics of the municipality-level NO_2_ and the environmental variables is given in Table 1.

**Table 1:** Municipality-level descriptive statistics of environmental variables for South Africa.

	Minimum	Maximum	Mean	Median	Standard Deviation	Coefficient of variation (%)
NO_2_ (mol/m^2^)	0.0000079	0.00022	0.0000306	0.000019	0.000032	104.58
EVI	0.061	0.4	0.227	0.223	0.075	33.04
LST (K)	295.6	309.9	302.6	302.3	3.0	0.1
AOD	0.085	0.176	0.125	0.125	0.022	17.6

#### 2.2.2 Social data

Population density data was downloaded from (https://openafrica.org/) for the year 2016. The dataset provides the geographical code, spatial extent, population count and population density of each municipality. The datasets representing municipality-level variables on household-level energy use, sex, age and dwelling types were acquired from STATISTICS SA (http://superweb.statssa.gov.za/webapi/jsf/tableView/tableView.xhtml). These datasets were collected through a national-scale community survey conducted in 2016. Such a large-scale survey is carried out by gathering household social data that are aggregated to the municipal administrative level. The 2016 datasets, therefore, represented the closest (in terms of time) to the NO_2_ data at the municipality level. These data show the count of households belonging to each variable. Household energy use variables included coal, wood, electricity and paraffin for both cooking and space heating. The dwellings variable consists of stand-alone dwellings, townhouses, flats or apartments, a cluster of buildings in a complex, and informal settlements. Residence in any of these dwelling types can be linked to economic status, with well-off households affording stand-alone houses while low-income earners are forced to live in smaller and informal settlements [60]. Dwelling type can influence household energy source and demand [61] and therefore, it is relevant to explore how all dwelling types relate to NO_2_ emission. The age variable included the number of people up to 69 years of age divided into 13 categories, each with a 5-year range. Maintaining the narrow-range age categories was preferred since it is unknown if such groups can influence NO_2_. It is believed that analysis using such detailed categories for as long as possible does not compromise what would be obtained using a more generic category, as nearby categories would return similar results if similarities existed. The data on sex included the numbers of males and females of each municipality. All the above social data represented the year 2016. These data were, therefore, merged with the spatial data of South Africa’s municipality map that was published in 2016 [53]. The descriptive statistics of all the social variables are summarised in Table 2. In total, 35 variables, including three environmental and thirty-two social variables, were included for NO_2_ estimation.

**Table 2:** Municipality-level summary statistics of social variables for in South Africa.

	Social Parameter	Mean	Median	Standard deviation	Minimum	Maximum
Energy use for cooking	Electricity Cooking	3 718.1	1 469	8 947.5	156	67 732
	Gas Cooking	122.2	60	271.4	3	3 147
	Paraffin Cooking	233.7	53	836.5	1	9 352
	Wood Cooking	500.1	129	813.9	2	5 219
	Coal Cooking	15.4	1	50.3	0	365
Energy use for heating	Electricity Heating	2 878.8	1 008	7 716.6	86	62 812
	Gas Heating	48.9	16	143.5	0	1 276
	Paraffin Heating	271.5	34	745.4	1	5756
	Wood Heating	560.8	264	680.7	4	3 432
	Coal Heating	45.9	4	188.1	0	2364
Dwelling type	Flats or Apartments	134.6	15	537.8	0	4691
	Cluster In Complex	29.9	3	122.6	1	973
	Townhouse	28.7	4	117.7	0	1172
	Informal Dwellings	309.5	60	1 040.7	0	8232
	Formal Dwellings	3 032.6	1 289	6 336.5	157	47 482
Population density	Population Density (km^2^)	111.3	40.1	300.4	0.4	3,008.8
Gender group	Male Sex	2 319.8	1 065	5 518.4	83	42996
	Female Sex	2 288.7	1 126	4 478.9	81	33 790
Age group	Age 0–4	1 528.9	874	2 536.0	37	19 161
	Age 5–9	1 612.6	920	2 566.9	45	18 714
	Age 10–14	1 451.4	837	2 259.4	37	16 257
	Age 15–19	1 443.4	819	2 403.1	31	16 853
	Age 20–24	1 410.5	682	2 924.2	29	21 757
	Age 25–29	1 354.5	583	3 098.4	35	24 100
	Age 30–34	1 217,2	512	2 875.8	27	22 278
	Age 35–39	1 015.9	436	2 442.6	22	19 035
	Age 40–44	907.2	415	2 089.9	28	16 040
	Age 45–49	803.0	369	1 750.7	35	12 990
	Age 50–54	729.5	347	1 495.8	26	10 961
	Age 55–59	626.3	316	1 223.6	24	8 944
	Age 60–64	504.3	258	945.6	21	6 782
	Age 65–69	389.8	199	712.2	16	5 383

### 2.3 Pre-processing

Since this study used a linear regression model to associate the NO_2_ and socio-environmental variables, the variables had to be in a continuous numerical scale. Therefore, each social variable data was converted from count to continuous scale using the areal spatial interpolation technique [62]. The technique uses a kriging-based interpolation suitable for data collected within areal extents such as municipalities used in the current study. The approach is specifically skilled in accounting for the area of each polygon (i.e., municipality), thereby ensuring the proportional treatment of different-sized municipalities and counts of social data. The interpolated continuous data was subsequently aggregated by the municipality since the final regression analysis was done at such a scale. The plot-level median value was best compared with the original count data (i.e., Pearson correlation coefficient, *r* = 0.92–0.99) for each variable and, therefore, was used to derive the municipality-level social variables data. The spatial distributions of municipality-level social data used in the study are shown in Fig 2, while selected statistics of those data are summarised in Table 2. Higher concentrations of the social data values are found mostly in the eastern part of the countries, although there were variations among the variables justifying their inclusion in the study. These distributions largely coincide with the distributions of NO_2_ column density in the country.

Although all the variables were converted into continuous scale, they still had different measurement units. As a result, all the response and explanatory variables were converted to a standard scale before building the regression model. In the present study, a standard score (also referred to as Z-score) was applied to each variable. The Z-score is computed by subtracting each value from the mean of all values and subsequently normalizing it by the standard deviation of all values. This process produces a value range with a mean of zero and a standard deviation of one, making the data suitable for MGWR modelling (Section 2.4). Although a linear model built on the global ordinary least square regression requires normality of data distribution, the MGWR does not need to honour a distribution pattern since the localization at different scales limits the sample sizes for each model. Standardization of variables is advantageous if the original input variables are measured in different units—as is the case in this study. The resultant parameter estimates (i.e., coefficients) derived from the scaled variables allow for a direct comparison of the influence of all explanatory variables. The outcome of the Z-score is a value range with a mean of zero and a standard deviation of one.

$$Ζ=\frac{\left(Χ-\mu \right)}{\sigma }$$ (1)

where Χ represents the value of the observed variable, *μ* is the mean value of the dataset and *σ* represents the standard deviation of the dataset [63].

### 2.4 Statistical analysis

The present study used a regression analysis to estimate NO_2_ column density using the environmental and social variables as predictors. The Geographically Weighted Regression (GWR) is a non-stationary method that models a spatially varying relationship between a dependent variable and a set of explanatory variables [64]. Unlike the standard global linear regression that builds a single model using all the data in a study area, the GWR creates multiple models in different localities of a study area as long as sufficient variation exists among localities [64]. Thus, the method builds on Tobler’s first law of geography, which states that closer features on the Earth’s surface are more related than further ones [65]. The variation of all the data across space makes the GWR regression suitable for the present study similarly to previous studies [29, 66, 67]. The GWR is quantified as:

$$Y_{i}=a_{0}\;\left(U_{i},\;V_{i}\;\right)\;+\;\sum_{\kappa }a_{\kappa }\;\left(U_{i\;},V_{i}\;\right)X_{i\kappa }\;+\;{ℇ}_{i}$$ (2)

where $\left(U_{i},\;V_{i}\right)$ characterizes the geographical coordinates of the point i and *a*_*κ*_ $\left(U_{i},\;V_{i}\right)$ represents the continuous function *a*_*κ*_ (U,V) at point i [64]. The GWR is considered a fixed-scale model that utilizes a single bandwidth parameter that determines how distance decay is used to weigh nearby data around each location’s coefficients [68]. This single bandwidth assumes that all relationships between the dependent and independent variables occur on an equal spatial scale [68].

The Multiscale Geographically Weighted Regression (MGWR) is an advanced GWR allowing bandwidth determination of each variable independent of other variables [69]. As such, the MGWR recognizes the variation in the influence zone that individual explanatory variables may have on the response variable. The formula for the MGWR is given as:

$$Y_{i}=\sum_{j=0}^{m}\beta_{bwj}\left(U_{i},\;V_{i}\;\right)\;X_{ij}+{ℇ}_{i}$$ (3)

where bwj in $\beta_{bwj}$ represents the bandwidth used to calibrate the *j*th conditional relationship [69]. The MGWR model is calibrated using the back fitting algorithm, which uses the expected log-likelihood method for parameter estimation [69]. In implementing the MGWR, the neighbourhood to build an optimal model for each municipality was determined using the number of neighbours. This decision is justified considering the variation in spatial size of municipalities; if distance, rather than number of neighbours, were used, there would be no guarantee of multiple members in a neighbourhood around large-sized municipalities. For each predicting socio-environmental variable, the neighbourhood size (i.e., number of neighbours) was optimized by building models iteratively and selecting the best one that returned the smallest Corrected Akaike’s Information Criterion (AICc). All analysis was performed in ArcGIS Pro 3.1.

## 3. Results

### 3.1 Local bivariate relationships between NO_2_ column density and socio-environmental variables

**Fig 3:**
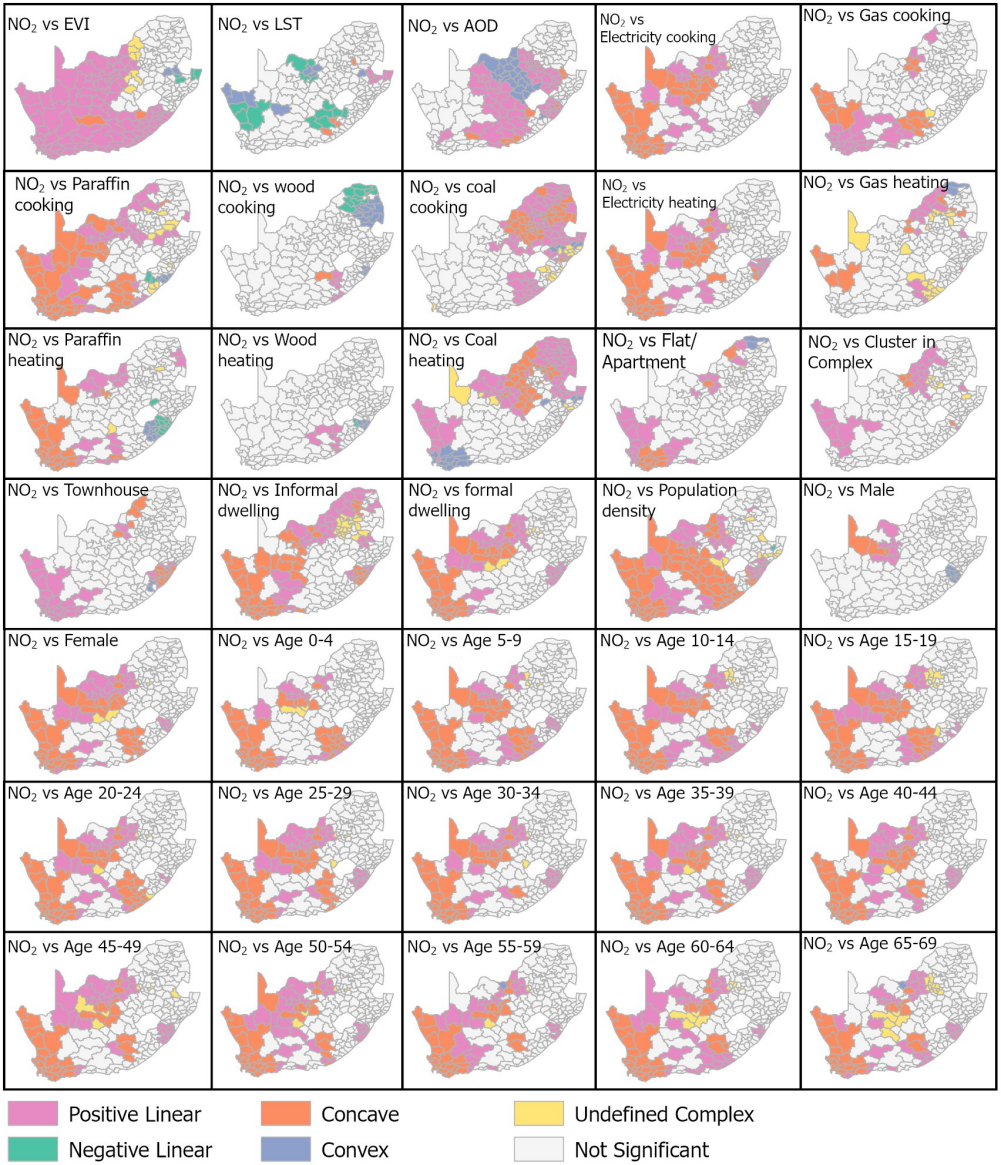
A bivariate relationship between NO_2_ and each explanatory socio-environmental variable across South African municipalities.

Exploring the spatial variation in the type of relationship between each variable and NO_2_ across the country is beneficial before running the MGWR. Fig 3 shows spatial variations in the significance levels and types of the bivariate relationships. The environmental variables EVI and AOD had a positive linear relationship with NO_2_ in nearly all the municipalities in the western part of the country reaching up to R^2^ of 0.83. The AOD, in particular, showed this type of relationship for a more significant part of the country than EVI. The significant relationships in the western portions of the country coincide with relatively low NO_2_ column density. Notably, in the eastern part of the country, where NO_2_ was relatively high, EVI and AOD had a non-significant or complex relationship with NO_2_. The LST was inversely correlated with NO_2_ (maximum R^2^ = 0.79) predominantly in the northern part and some pockets in the west and south-eastern parts of the country. In the rest of the country, the LST had no significant relationship with NO_2_.

The relationship between energy sources for cooking and NO_2_ showed clear spatial patterns, with significant influence (i.e., R^2^ up to 0.79) observed mainly in the western part of the country (Fig 3). Moreover, a concave relationship was noted in the far western parts, while the immediate adjacent parts showed a linear positive influence on the increase of NO_2_. Wood usage had the lowest significance in estimating NO_2_ out of the five energy sources for cooking. The relationship between coal usage for cooking and NO_2_ was markedly significant in the eastern part of the country. The relationship between energy consumption for heating purposes and NO_2_ had largely similar spatial patterns of significance as the ones observed for cooking purposes. Two distinct differences can be seen with more significant influence in paraffin usage for cooking than for heating, as well as the more significance of coal for heating (maximum R^2^ = 0.8) than for cooking (maximum R^2^ = 0.7) in the western part of the country. The relationship between the number of households and NO_2_ shows variation for each dwelling type, with a positive linear relationship being the most common type. In addition, a non-significant relationship between all dwelling types and NO_2_ was observed mainly in the central parts of the country. Flats/apartments (maximum R^2^ = 0.65), and formal (maximum R^2^ = 0.68) and informal (maximum R^2^ = 0.70) dwelling types had the most significant relationship with NO_2_. The formal and informal dwelling type had significant but non-linear (concave) relationships with NO_2_ in the far western parts of the country.

Population density exhibited a significant relationship (maximum R^2^ = 0.82) with NO_2_ for a large part of the country, with most of the relationship with NO_2_ being positive linear or concave along the western and northwestern parts of the country (Fig 3). The relationship was generally insignificant in the central and eastern parts of the country for population density (Fig 3). The significance of the number of females (maximum R^2^ = 0.65) followed the same pattern as the number of males (maximum R^2^ = 0.77) but in a smaller number of municipalities than in the case of males. Regarding the influence of age groups, the most significant relationship with NO_2_ was observed in the western half of the country in all age groups (Fig 3). Strikingly, the relationships were non-linear (concave) in the far western part of the country for all age groups.

### 3.2 NO_2_ prediction using socio-environmental variables

**Fig 4:**
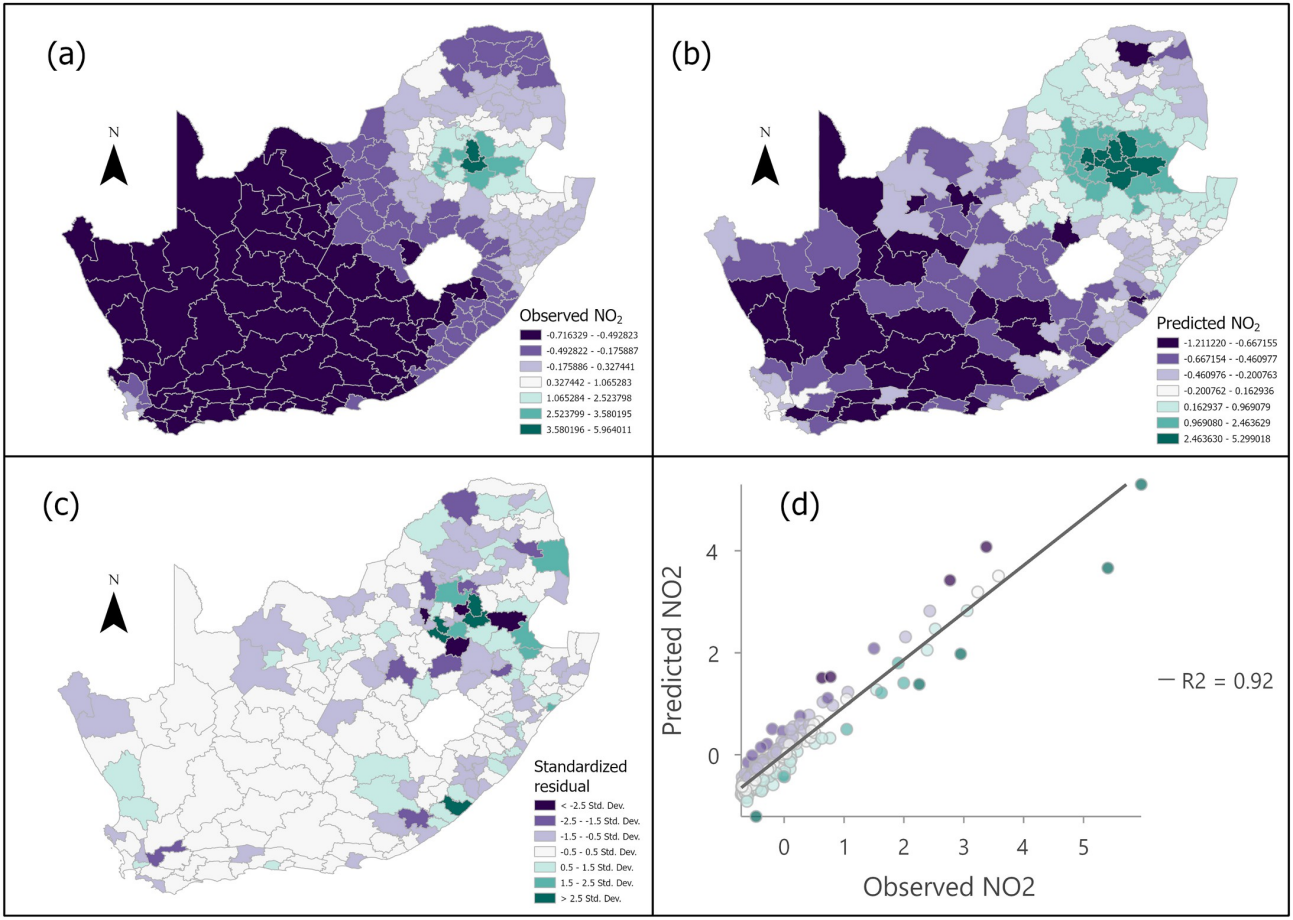
The annual spatial distribution maps of standardized NO_2_ vertical column density; (a) Observed standardized NO_2_, (b) Predicted standardized NO_2_, (c) Standardized residual map, (d) Scatterplot of the relationship between the observed and predicted standardized NO_2_.

Fig 4 shows the results of annual average NO_2_ distributions across South Africa for the period December 2018 to November 2019. The results represent estimations achieved using all socio-environmental variables (n = 35) as predictors in MGWR. The observed NO_2_ ranged between -0.72 and 5.96 mol/m^2^ (Fig 4a) while the predicted values ranged between -1.21 and 5.30 (Fig 4b). This indicates the overall agreement between the observed and estimated NO_2_. Furthermore, the MGWR predictions reflect the spatial patterns with higher and lower NO_2_ in the northern and western parts of the country, respectively. The municipalities with the lowest prediction errors were concentrated mostly in the western part, with standardized residuals of -0.5 to 0.5 (Fig 4c). There were 95 and 118 municipalities with over- and under-estimated NO_2_ column density, respectively. However, a global autocorrelation analysis of the errors using the Moran’s I index [70] showed a random distribution across the country (a low z-score of -0.902563; *p* = 0.366758), indicating the lack of spatial pattern or bias in the estimation error. On the other hand, each social and environmental variable showed significant global clustering (z-score ranging from 3.388443 to 32.678918; *p* < 0.001). The high correlation between the observed and predicted NO_2_ column density at R^2^ = 0.92 shows the predicting capability of the socio-environmental variables considered in the study (Fig 4d). The strongest correlations were observed mainly when standardized NO_2_ values were low, with approximately 1 mol/m^2^ or less, although even the higher column density was estimated well.

**Fig 5:**
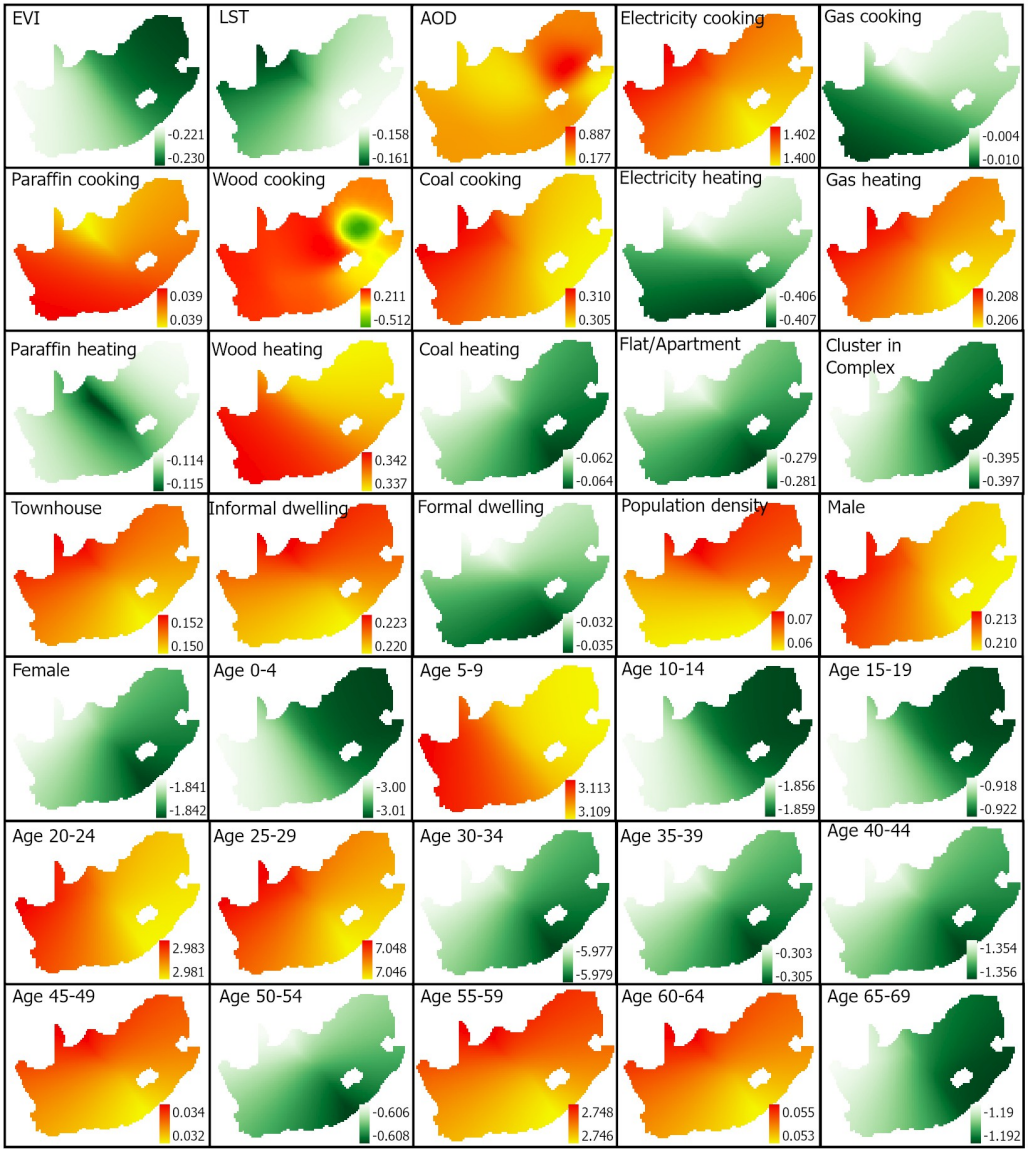
Spatial distributions of coefficients (parameter estimates) of variables used to predict NO_2_ vertical column density. Variations of green colour show the negative (opposite) influence of a variable on NO_2,_ while the yellow-to-red colour scheme shows the positive influence on NO_2_.

The spatial distribution of each predictor’s influence on NO_2_ is shown in Fig 5. The two environmental variables, EVI and LST, have an inverse influence on NO_2_ throughout the country, with higher values of the two variables resulting in lower NO_2_ column density. Comparing the two environmental variables, the EVI (-0.221 to -0.230) has more impact than LST (-0.158 to -0.161). Furthermore, the EVI’s influence is higher on the eastern side of the country than in the west, while the opposite is true for the LST. AOD had a direct influence on NO_2_ levels across the country (0.177 to 0.887), with higher impacts seen in the northeastern parts than in the rest of the country (Fig 5).

Energy usage for cooking also directly influences NO_2_ except for gas-based cooking, which showed an inverse influence on NO_2_ as seen in Fig 5. Another exception is observed in wood usage for cooking which had an inverse impact of NO_2_ in a pocket in the north-eastern part of the country. Apart from wood usage for cooking, the variations in the influence of each energy usage across the country were low. A comparison of the influence of the energy sources for cooking indicates that electricity has the most influence on NO_2_ increase (i.e., 1.400 to 1.402), whilst gas (-0.004 to -1.010) has the lowest influence. Energy usage for heating directly influences NO_2_ when gas and wood are used but has an inverse impact on electricity, paraffin and coal sources. The impacts of electricity and coal usage for heating ranked the most and least influential, respectively, on the estimation of NO_2_. Like the spatial distributions of energy sources for cooking, the influence of each energy source for heating showed relatively low variations across the country.

Among the dwelling types, flat/apartments (-0.281 to -0.279), clusters in complex (-0.397 to -0.395) and formal dwellings (-0.035 to -0.032) reduced NO_2_ levels throughout the country while the opposite influence was observed in townhouses and informal dwellings with coefficient values ranging between (0.150 to 0.152) and 0.220 to 0.223), respectively (Fig 5). Regarding the influence of population on NO_2_ prediction, the results show that population density and the number of males affect NO_2_ directly, while the number of females has the opposite effect on NO_2_. Of the population data, the number of females had the most important contribution (-1.840 to -1.841) to the NO_2_ level, followed by the number of males (0.210 to 0.213). The number of people by age category showed mixed levels of influence on NO_2_ column density, with age groups of 5–9, 20–24, 25–29, 45–49, 55–59 and 60–64 showing a direct influence while the rest had a negative influence on NO_2_. The younger and mid-age groups (up to 44) than the rest had more impacts on NO_2_ levels, although the spatial variations of impacts for each social variable were relatively low.

## 4. Discussion

### 4.1 NO_2_ correlation with individual socio-environmental variables

The key objective of the study was to assess the link between socio-environmental variables and atmospheric NO_2_ column density distribution in South Africa. Each socio-environmental variable correlated with NO_2_, with the environmental variables of EVI and AOD showing the most correlations across the country (Fig 3). The strong association of AOD with NO_2_ level is not surprising considering that aerosols comprise all suspended particles, including NO_2_. The inverse correlation between EVI and NO_2_ is generally known; however, the present study showed an increase in NO_2_ with an increase in EVI, particularly in the western part of the country. The western part is characterized by arid conditions and thus less vegetated than the east [71]; this can also be confirmed by the EVI distribution in Fig 2. This suggests that the vegetation did not have a sufficient amount to influence the NO_2_ emission levels in the area. This observation is consistent with a study by [72] in India, which indicated that population density and the number of people in the younger age groups (up to age 24) also contributed to the increase of NO_2_ for a large part of the country. Population density effects on air pollution especially in urban setups is linked to the extent of mobility and energy usage [24]. The younger populations’ higher influence on NO_2_ is expected, given that this group is associated with commuting to schools, which contributes to transport-driven pollution [73]. A linear positive relationship was the most common correlation, followed by a concave relationship, while wood usage for cooking and heating purposes showed significant correlations with NO_2_ for a limited number of municipalities. The concave relationships indicate the negative effect of a variable on NO_2_ up to a certain NO_2_ level, beyond which the relationship becomes positive.

### 4.2 Capability of socio-environmental variables to predict NO_2_

The MGWR-based modelling that used all socio-environmental variables as explanatory variables returned a high R^2^, i.e., 0.92 (Fig 4). Low errors distributed randomly, as opposed to dependence on space, across the country confirms the strength of the prediction. This is despite the clustering of values for each social and environmental variable, with high values observed mainly around major urban areas for most of the variables. The unbiased spatial distribution of the error justifies the use of GWR modelling that computes parameter estimates for each locality [64]. Furthermore, the advantage of using the improved MGWR that determines the scale of each explanatory variable independently [69] was evident in this study. This can be verified by the fact that individual factors correlated with NO_2_ differently (Fig 3) and thereby have varied influence zones (spatial scales) on NO_2_. Regarding the spatial distributions of NO_2_, high values were predicted in the north-central or north-eastern part of the country, matching the observed map (Fig 4). The match of this distribution with that of the AOD is notable. Most of South Africa’s power plants (Fig 2) and industrial activities such as mining are found in the north-central or north-eastern parts [74, 75]. These activities emit byproducts that elevate the concentration of AOD, which includes NO_2,_ among other particulate matters [75, 76]. Our findings are compared to a global air pollution modelling study, which suggested a positive correlation between NO_2_ and AOD is observed across the world due to similar emission sources [77]. Moreover, [77] observed this relationship in regions of high traffic density which is a variable that was overlooked in this research. The significance of AOD on NO_2_ in this study is evident from the large range (0.177–0.887) of the coefficients compared to the lower and narrower ranges of EVI and LST (Fig 5).

Among energy sources and uses, electricity consumption for cooking purposes had the biggest contribution to NO_2_ levels (Fig 5). The limited variation in the influence of electricity usage can indicate the homogenously high demand for it across the country, suggesting the gloomy outlook in reducing NO_2_ pollution. The use of wood for cooking had a mixed effect on NO_2_ levels, showing a reduction effect only in the north-central part of the country, compared to its increasing effect in the rest of the country. The increase in the number of people residing in flats/apartments and in clusters within complexes was associated with a reduction in NO_2_ levels. Such dwelling types share restricted management of resources, including energy utilization; thus, their emission contribution remains limited [78]. In comparison, informal dwellings and townhouses exercise more relaxed freedoms in using energy for different purposes [79]. Although informal settlements in South Africa continue to struggle with a lack of service deliveries, many of them access electricity through illegal connections thus adding pressure to coal-powered stations [79, 80]. Because such dwellers do not pay for electricity services, there is a potential of unrestricted electricity usage in such dwelling types. Since this study found positive association between NO_2_ emission levels and electricity consumption, it is critical for policymakers to consider the management of such dwelling types.

The female population group had the biggest impact on NO_2_ pollution and was associated with the reduction in pollution (Fig 5). This contrasted with the impact of the male group that was associated with increasing pollution levels followed by the population density variable. The finding regarding the reducing effect of the female population group on NO_2_ emission is encouraging, considering that most of the household activities (in most African contexts) are carried out by this group. Moreover, studies have suggested that women tend to be more informed about climate change and show concern for air pollution compared to their male counterparts [81, 82]. This difference in perception on air pollution could justify the observation made by this study when the influence of gender on NO_2_ is concerned. The observed results suggest their wise consumption of energy for cooking and space heating since these activities heavily rest on them. This study may indicate that this strategy has been successful in the sense of greatly lessening the detrimental effects of males and population density variables with regard to their positive relationships with NO_2_ (Fig 5). The feedback effect of the NO_2_ pollution reduction on the female group should also be underlined, as women are generally the main victims of pollution due to their immediate exposure to domestic pollution sources [83]. Lower NO_2_ pollution associated with increase in the female population is therefore welcomed as it may contribute to improvement in air quality and reduce its health effects. Although the population density proved to be the least significant contributor of pollution, its significantly high contribution in the eastern than in the western part of the country is noteworthy (Fig 3). This is attributed to the fact that the eastern part, that includes the economic hub of the country, such as the Gauteng province as well as the agriculturally rich provinces, has a higher population density than the western part. This, in turn, adds to the increased consumption of industrial products and energy resources that lead to NO_2_ emissions. Ryu *et al*. [84] found similar results in a study conducted in South Korea of high NO_2_ concentrations in the metropolitan areas compared to rural areas where population density is lower and are less industrialized. Furthermore, a worldwide investigation by [77] reported similar results showing a direct relationship between population density and NO_2_, which specifically explained the variance of NO_2_ mostly in Asia where population density is high.

Similarly, the high NO_2_ level in the north-central or north-eastern part distinctly matched the high number of people dwelling in townhouses and clusters in complexes. Again, this is attributed to the prominence of the many dwelling types in the area to accommodate the large population participating in the region’s multitude of economic and academic activities [24]. However, it is important to take notice of other areas of the country with high concentrations of the other social variables in the eastern and western parts, in addition to the central part, but with low NO_2_ levels. This suggests that the pollution in those areas can be linked to variables that were not included in the study, or the impacts of the social variables were limited by environmental variables such as the EVI and AOD. Age of people had a mixed effect on NO_2_ levels, with certain groups linked to more pollution than others. The mid-age group’s (20–24 and 25–29) association with increased NO_2_ levels is noteworthy since this group has one of the largest population sizes. Similar findings were reported by [85] who showed that the working-class age group (20–34) had a positive link with air pollution in 17 developing countries as a result of transit to work. Tarazkar *et al*. [86] also indicated that individuals in the labour force consume more energy than children and the old population, and they also release more greenhouse gases as a result of higher production activity. Given the high use of transportation expected from the mid-age group for purposes such as economic activities most likey increases the pollution, concurring with other studies that reached at the same conclusion. As a result, the pollution contributed by this age group should present a concern unless the main cause of the association is identified and addressed. The age groups 55–59 and 60–64 also contributed to NO_2_ levels, though to a lesser extent than the mid-age groups. Estiri and Zagheni [87] attributed a higher energy consumption among older population groups and predicted an increasing trajectory of the effect for the future in the face of warming temperatures and climate change that place more demand on energy.

### 4.3 Study significance, limitations and recommendation

The findings of the present study showed high accuracies of NO_2_ column density predictions could be achieved by using social and environmental variables as predictors. Although the study does not claim to represent a causality analysis, it provides strong evidence of the capability of socio-environmental variables to explain the variation in NO_2_ levels. This capability can support environmental auditing programmes such as air quality monitoring and greenhouse gas emission accounting efforts. The monitoring and maintaining of clean air fit into various Sustainable Development Goals (SDGs) of the United Nations [88]. These include SDG Target 3.9.1, which focuses on the reduction in air pollution caused deaths and illnesses; SDG Target 7.1.2 which strives for access to clean energy in homes; and SDG Target 11.6.2, which promotes the reduction of the environmental impact of cities by improving air quality. One of the benefits of successfully predicting pollution using many social variables lies in the fact that all the pollution drivers can be managed given available resources and efficient management strategies. Even the environmental variables considered in the modelling exercise are largely influenced by anthropogenic activities, making them fall within the scope of social decision-making processes. Regarding the modelling technique, the MGWR approach proved highly successful in reducing spatially unbiased estimation errors. This approach is suitable when the focus area extent is large and variations due to location exist, as shown in the present study. The results revealed a varying level of influence of each socio-environmental variable across the country. Intervention efforts to reduce pollution by targeting these variables should, therefore be customized by location to invest resources efficiently.

Although the findings of the study were promising, future studies could improve on it as follows. Only three environmental variables believed to affect NO_2_ column density were used in the study. Adding more environmental variables, such as other atmospheric variables and climatic data can make the prediction robust and resistant to fluctuations of certain variables. The social variables used in the study were limited to rather outdated statistical data on household-level energy consumption, population density, sex proportions, dwelling types and age distributions. Additional and up-to-date data such as energy use by age, gender, and dwelling type carry more specific information on energy consumption pattern of a community, and thus, including such data can strengthen the prediction. Furthermore, data on transport would certainly improve the modelling accuracy as such dataset remains one of the major drivers of NO_2_ and other atmospheric pollutions [17, 72]. From the modelling perspective, it was opted in the study to use individual variables in the MGWR. The use of several variables in a model can suffer from collinearity among variables, resulting in an unnecessarily complex model. Such complexity can be reduced using different techniques one of which is Principal Component Analysis which combines the variables to generate a reduced number of uncorrelated explanatory variables. However, such an approach must be explored cautiously as it can result in removing information from the original variables. Another approach that can be explored to produce a robust pollution prediction model is using robust machine learning algorithms that do not require honouring statistical preconditions (e.g., data distribution) [89].

## 5. Conclusion

The impact of social and environmental factors on NO_2_ pollution remains one of the major challenges to achieving clean air goals. This study aimed to show the link between socio-environmental variables and atmospheric NO_2_ levels across South Africa using data obtained from remotely sensed sources and national population surveys. Results of the MGWR showed the explanatory power of socio-environmental variables to NO_2_ variations with an overall R^2^ of 0.92. The model’s accuracy is confirmed explicitly by the estimation errors that were not only low but also distributed randomly, indicating the unbiased prediction capability of the model. This represents a crucial success of the prediction, considering that individual explanatory factors varied with space. From the environmental variables, the AOD proved to be the major contributor to pollution while the EVI partially negated the impacts of AOD, providing the evidence to push for a greener environment. Electricity and coal usage for cooking and wood for space heating were most influential in the increase of NO_2_ compared to the other energy sources and uses. Targeting these energy sources and adopting environment-friendly sources and consumption can reduce pollution levels.

Dwelling type also had a significant impact on group residences, including clusters-in-complexes and apartments, reducing the pollution amount. Given the continued population increase along with global climate warming, group residence is perhaps one of the best options since it limits the resource-consumption culture of humans. In contrast, informal settlements expectedly increased the pollution level which can be attributed to disorganized service delivery such as illegal power connections, unsustainable energy usage, and poor waste management and transport systems. A working model is therefore needed to improve informal settlements by providing platforms that reduce the impacts of the current resource usage on pollution. As expected, an increase in population density coincides with an increase in NO_2_ pollution due to the intense use of energy and transport per given area. Reducing the pollution problem in densely populated areas requires a combination of intervention measures compared to what would be needed to mitigate the impacts of other factors. The impact of the female population in reducing NO_2_ pollution is one of the most encouraging findings of this study. Females carry most of the burdens in society and yet proved to be efficient in resource utilization to reduce pollution. However, the positive contribution of females is undermined by males, who showed a direct correlation with the elevated pollution levels. The effect of population age groups on NO_2_ pollution was mixed, with the mid-age group (20–24 and 25–29) being the main cause of increased pollution. These age groups make up a significant proportion and thus must be given greater attention when planning efforts to reduce NO_2_ pollution. While the study preferred to maintain the use of several factors as predictors of NO_2_ pollution, it is important to note the potential correlation among some variables that could result in overfitted models. This should be explored in future studies to reduce predictors and develop parsimonious models using only significant variables. Alternatively, an approach such as the principal component analysis that reduces predictors by extracting unique information from several variables can be explored. Lastly, the MGWR modelling yielded prediction characteristics that varied across space. Such spatial variation informs location-specific intervention measures as opposed to a generic, national-scale intervention strategy that not only may prove inefficient but also leads to wasteful expenditure of resources.
